# Lifecourse socioeconomic trajectories and C-reactive protein levels in young adults: Findings from a Brazilian birth cohort

**DOI:** 10.1016/j.socscimed.2009.12.014

**Published:** 2010-04

**Authors:** Aydin Nazmi, Isabel O. Oliveira, Bernardo L. Horta, Denise P. Gigante, Cesar G. Victora

**Affiliations:** a Food Science and Nutrition, California Polytechnic State University, 1 Grand Avenue, San Luis Obispo, CA 93407, USA; b Federal University of Pelotas, Pelotas, Brazil

**Keywords:** Brazil, C-reactive protein, Inflammation, Socioeconomic factors, Cohort studies, Gender, Lifecourse studies, Cardiovascular disease

## Abstract

Socioeconomic factors are associated with cardiovascular disease. C-reactive protein (CRP) is increasingly implicated as a candidate linking conventional risk factors and atherosclerosis. The impact of early- and later-life socioeconomic status (SES) on CRP levels has not been widely investigated and a handful of studies from high-income countries are inconsistent. We set out to examine the associations between lifecourse socioeconomic indicators (family income at birth, maternal education, family income at age 23 and own education) on CRP levels in young adults belonging to the 1982 Pelotas (Brazil) Birth Cohort Study (*n* = 5914). Early-life SES showed significant and graded associations with CRP levels at age 23 independently of later SES. For example, men with higher family income at birth showed higher CRP levels at age 23 (p = 0.001 for trend) and women with less educated mothers showed higher CRP levels (p = 0.01 for trend). Notably, differential directions of association between SES indicators and CRP levels between men and women were found. When adjusted for SES at age 23, men with the lowest family income at birth showed 42% lower CRP levels when compared to men in the highest family income group (−42; 95% CI: -60,-16). In contrast women born to the least educated mothers had the highest CRP levels (35; 95% CI -2, 86). In both sexes, adiposity accounted for the overwhelming majority of the associations between SES and CRP levels. Sex and gender roles specific to middle-income countries, socio-cultural and environmental conditions that may impact adiposity, and the level of epidemiological transition may be key factors that are linked to the associations between lifecourse SES and CRP levels. Public health strategies aimed at decreasing the burden of cardiovascular disease in middle-income settings, in addition to highlighting the risks associated with adult obesity, should not overlook the wide-ranging impacts of lifecourse social determinants.

## Introduction

Socioeconomic factors are powerful determinants of cardiovascular disease (CVD) in high-income countries. It has been widely demonstrated that poorer and less educated individuals suffer significantly higher levels of mortality and morbidity associated with CVD than richer and more educated ones and that these patterns follow marked dose-response patterns (Cooper et al., 2000; Dalstra et al., 2005; Kaplan & Keil, 1993; Mackenbach, Cavelaars, Kunst, & Groenhof, 2000; Winkleby, Kraemer, Ahn, & Varady, 1998). Several of these studies have addressed potential pathways through which low socioeconomic status (SES) may lead to CVD, reporting that lifestyle factors such as diet, exercise and smoking fail to completely account for differences in outcome. It is, therefore, feasible that poorly understood or unknown biological pathways play important roles. Moreover, distribution patterns of risk factors may be different between men and women in low and middle-income countries (McLaren, 2007; Monteiro, Moura, Conde, & Popkin, 2004).

C-reactive protein (CRP), a non-specific indicator of systemic inflammation associated with the acute phase response, has been extensively studied as a potential candidate linking conventional risk factors and the atherosclerotic process (Danesh et al., 2004; Koenig et al., 1999; Pepys & Hirschfield, 2003; Ridker, Buring, Shih, Matias, & Hennekens, 1998; Ridker, Cushman, Stampfer, Tracy, & Hennekens, 1997). C-reactive protein in highly-sensitive assays has been shown to predict coronary events, as well as type 2 diabetes mellitus and sudden death associated with CVD in prospective observational studies, mostly in middle-aged adults (Cesari et al., 2003; Freeman et al., 2002; Hu, Meigs, Li, Rifai, & Manson, 2004; Pepys & Hirschfield, 2003; Ridker et al., 1998; Thorand et al., 2003). Mendelian Randomization studies suggest that CRP levels have a genetically heritable component, but that CRP is not likely causally related to CVD outcomes. Although debate continues as to the exact role of CRP in the atherosclerotic process (Lloyd-Jones, Liu, Tian, & Greenland, 2006), the Centers for Disease Control and the American Heart Association suggest that chronically elevated CRP levels should be considered in conjunction with conventional risk factors for CVD (Pearson et al., 2003).

In high-income countries, individuals of higher socioeconomic standing have lower CRP levels compared to those with lower SES (Alley et al., 2005; Jousilahti, Salomaa, Rasi, Vahtera, & Palosuo, 2003; St. J O'Reilly et al., 2006). A recent systematic review of 25 population-based studies concluded that individuals with lower SES were more likely to have elevated CRP levels than those with higher SES even after adjustment for a number of covariates including adiposity (Nazmi & Victora, 2007). This suggests that CRP may be one of the factors that mediate the association between low SES and CVD. This review located only one study from a middle-income population (Turkey), and few authors attempted to disentangle factors that confound the SES–CRP association from those that may act as possible mediators (Kivimaki et al., 2005; Lawlor, Davey Smith, Rumley, Lowe, & Ebrahim, 2005; Onat et al., 2001).

There is compelling evidence from high- and middle-income countries that early-life socioeconomic conditions may contribute to CVD risk (Barker, 1994; Barros & Victora, 1999; Monteiro, Victora, Barros, & Monteiro, 2003; Nazmi, Huttly et al., 2007; Yajnik et al., 2003). The impact of early-life SES on CRP levels has not been widely investigated, and a handful of studies from high-income countries show somewhat inconsistent findings (Gimeno et al., 2008; Pollitt et al., 2007; Tabassum et al., 2008). No studies have examined these associations in a representative sample in a middle-income setting, where conventional risk factors for CVD are known to have different distributions among the population compared to high-income countries.

It is unclear exactly what differences can be expected in terms of the associations between SES indicators and CRP levels when examining the low, middle, and high-income contexts. The numerous studies referenced above from high-income settings are relatively consistent in their findings. However, studies from low and middle-income countries are far fewer and are urgently needed, especially as the developing world is home to the majority of global CVD incidence (WHO, 2006). Findings in the middle-income context suggest that low SES is associated with higher levels of obesity in women but lower levels in men (González, Nazmi, Yudkin, & Victora, 2009; Monteiro, Conde, & Popkin, 2007; Monteiro et al., 2004). Data on SES and inflammation in the context of obesity in middle-income settings, however, is not available.

To address some of these gaps in the literature, we examined the associations between lifecourse socioeconomic indicators on CRP levels among young Brazilian adults. Specifically, we used data gathered at birth and at age 23 years from the prospective 1982 Pelotas Birth Cohort Study.

## Methods

The 1982 Pelotas birth cohort recruited over 99% of births occurring in the city that year. Mothers who gave live birth in one of the city's hospitals and who lived in the urban area of the city were included (*n* = 5914). Newborns and their mothers were weighed and a questionnaire was applied. Cohort members have been prospectively followed up over the years in numerous visits; the main phases being at one, two, four, 15, 18 (males only), 19 and 23 years old. Information on follow up methods, sampling fractions and variables collected has been detailed elsewhere (Barros, Victora, & Vaughan, 1990; Cesar G. Victora & Barros, 2006; Victora et al., 2003).

In 2004–2005, when the cohort was approximately 23 years old, a citywide census of all 98,000 households in the urban area of Pelotas was performed in an attempt to locate members of the original cohort. Additional strategies included searching at the last known address, contacting relatives of the cohort members and registration with the universal health system. Trained interviewers applied questionnaires from October 2004 to September 2005.

Several socioeconomic and demographic variables from 1982 and 2004–2005 are available. Self-described race/ethnicity of participants was categorized as white, mulatto, black, Indigenous and Asian. Family income data at birth and at age 23 was categorized into groups representing ≤1, 1.1–3.0, 3.1–6.0, 6.1–10.0 and ≥10 monthly minimum wage units. Minimum wage was approximately 50 and 180 USD per month at birth and at the time of the 2004–2005 cohort visit, respectively. Number of years of formal education completed by the mothers in 1982 and cohort members in 2004–2005 was also collected and grouped in descriptive analyses as 0–4, 5–8, 9–11 and ≥12 years.

Body mass index (BMI) was measured using standardized methods for weight and height and calculated as kg/m^2^. Waist circumference was measured at the narrowest girth of the trunk or halfway between the costal margin and iliac crest. BMI and central obesity categories were defined using WHO cutoffs (WHO, 2000). Body mass index categories were: <18.5 kg/m^2^ underweight, 18.5–24.9 kg/m^2^ normal range, 25.0–29.9 kg/m^2^ overweight, and 30.0+ kg/m^2^ obese. Central obesity was considered as ≥94 cm for men and ≥80 cm for women. Smoking at least one cigarette every week was coded dichotomously for current smoking. A modified Block method was used to indicate fat and fiber intake (Block, Clifford, Naughton, Henderson, & McAdams, 1989). Alcohol consumption was categorized as “non-drinker”, “up to one drink per day” (equivalent to 350 mL beer, 150 mL wine or 30 mL liquor) or “>1 drink per day”. The long version of the International Physical Activity Questionnaire (www.ipaq.ki.se) was used to define sedentary behavior (<150 min of moderate intensity per week of activity during leisure time). The Self-Reported Questionnaire-20 (SRQ-20), which has been validated in Brazil, was applied and used to proxy psychological stress (Mari & Williams, 1986). Interviewer methods were standardized during four phases prior to and throughout the cohort visit. Other quality control procedures included re-evaluation of the questionnaire of 10% of the interviewees by study supervisors. Further details on behavioral and anthropometric variables collected and methods utilized in 2004–2005 are available (Nazmi, Oliveira, Gigante, Horta, & Victora, 2007).

During the 2004–2005 cohort visit, non-fasting venous blood was collected from volunteers and high sensitivity C-reactive protein was measured using an automated chemiluminescent immunoassay (Immulite, DPC/Siemens, Los Angeles, USA). The intra and inter coefficients of variation were 10 and 7%, respectively. C-reactive protein samples with results below the assay sensitivity threshold of 0.1 mg/L were converted to 0.05 mg/L for analysis.

### Statistical *analysis*

**Fig. 1:**
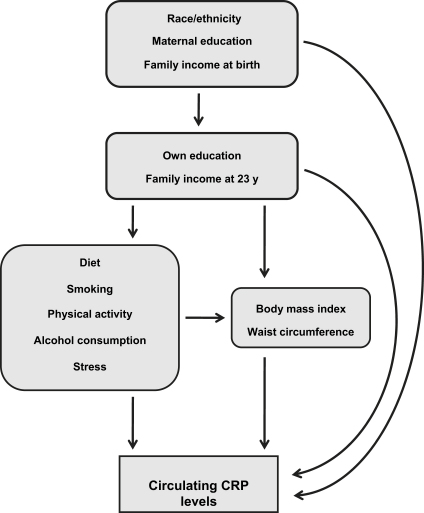
Proposed associations between socioeconomic indicators at birth and age 23 years with C-reactive protein (CRP) levels in young adulthood.

Analyses were based on a conceptual model defined *a-priori* (Fig. 1). As expected, there was evidence of effect modification by sex (see Results), all analyses were, therefore, stratified by sex. One-way ANOVA was used to test for sex differences in continuous variables from 2004 to 2005 (BMI and CRP levels) and chi-squared tests were used to test for differences in categorical variables (race/ethnicity and SES indicators at birth and at age 23; and percent central obesity; high/very high fat diet; low fiber intake; >1 alcoholic drink/d; sedentary at leisure; and high stress level).

Regression models used log-transformed CRP levels in analyses (due to skewed distribution) and are presented in exponentiated form to facilitate interpretation. Beta coefficients and 95% confidence intervals that appear in Tables 2 and 3 represent percent change in actual CRP levels compared to the reference category. *P*-values indicate tests for linear trend and point estimates reflect results using Wald tests for the above categories with the highest income and education groups as reference categories.

To elucidate the effects on the outcome at various levels of adjustment, lifecourse socioeconomic variables were first tested for associations with CRP levels in bivariate (unadjusted) analyses. Model 1 adjusted for sociodemographic variables (age, race/ethnicity) and SES variables at birth (family income at birth and maternal education). Model 2 further adjusted for SES variables at age 23 (family income at age 23 and own education), that may be influenced by SES at birth. Model 3 further adjusted for anthropometric and behavioral variables (BMI, waist circumference, smoking, fat/fiber intake, physical activity level, and stress) that may be influenced by the variables in models 1 and/or 2. BMI and abdominal circumference were entered as continuous variables, as was stress (SRQ-20) score. The remaining variables were entered in categorical format, as described above. In the last model of analysis, parity (number of children) in women was entered as a covariate, representing only difference between the models for women and men. Pregnant women (*n* = 93) and women using oral contraceptive therapy (*n* = 445) at the time of the blood draw were excluded from all analyses, as these groups have consistently been shown to have elevated CRP levels (Watts, Krohn, Wener, & Eschenback, 1991; Williams, Williams, Milne, Hancox, & Poulton, 2004).

The Federal University of Pelotas Ethical Committee approved all phases and aspects of the 1982 Pelotas birth cohort study. Verbal informed consent was obtained in 1982 and written informed consent in 2004–2005 for the questionnaires and blood draw.

## Results

Adding together subjects known to have died before the 2004 visit (*n* = 282) and those successfully interviewed (*n* = 4297) accounted for 77.4% of the original cohort. The mean age of those interviewed was 22.7 y (21.8–23.7) and 75% of individuals described themselves as white. CRP levels were assessed in 89% of interviewees; 1919 men and 1908 women. Individuals who did not have CRP measured had higher family income in 2004–2005, but were similar in terms of skin color, education (2004–2005), and anthropometric and behavioral variables to those who did. Excluding women who were either pregnant or using oral contraceptives at the time of the blood draw, median (IQR) CRP levels were 0.84 mg/L (0.38–1.98) and 1.68 mg/L (0.64–4.37) in men and women, respectively. The difference between the sexes was significant (*p* < 0.0001) but there were no differences in CRP levels according to race/ethnicity in either sex.

Distributions of socioeconomic indicators at birth and at age 23 for men and women, together with relevant covariables, are presented in Table 1. In both sexes, there were positive shifts in family income from 1982 to 2004 and in attained education level compared to maternal education. There were no differences in socioeconomic indicators at birth between the sexes. In 2004, cohort women were better educated on average than cohort men, but men had higher mean family income (both *p* < 0.001). Men had slightly higher BMI (*p* = 0.06), whereas significantly more women than men were centrally obese (27% and 10%, respectively). More than 60% of men and women had very high fat diets and nearly 70% had low fiber diets; no differences were observed between the sexes. More men consumed more than one alcoholic drink per day, whereas more women were sedentary at leisure and exhibited high self-reported stress levels (all *p* < 0.001).

Tables 2 and 3 show the associations between lifecourse socioeconomic indicators and CRP levels in men and women, respectively, in models 1, 2, and 3 according to the conceptual model, expressed in percent change (95% CI) in CRP levels compared to the reference category. *P*-values for trend are also shown.

### Results for early-life SES indicators (family income at birth and maternal education)

Unadjusted associations between early socioeconomic indicators and CRP levels were inconsistent between the sexes. In men, higher family income at birth was associated with higher CRP levels, whereas in women this association was not apparent. Higher maternal education, on the other hand, was associated with lower CRP levels in women and not associated in men. These significant associations were of similar magnitude. Men in the highest family income group at birth had 42% higher CRP levels than men in the lowest group, whereas women whose mothers had the least education had 43% higher CRP levels than those whose mothers had the most.

When controlled for age, race/ethnicity and for one another (model 1), associations with maternal education and family income at birth were slightly strengthened and retained significance with CRP levels in both sexes. These associations remained largely unchanged when further adjusted for family income at age 23 years and own education (model 2).

Behavioral and anthropometric variables were then included (model 3). In men, the association with family income at birth was slightly attenuated but still significant; men in the highest income category had 35% higher mean CRP levels than those in the lowest income group (*p* for trend = 0.007). Also in men, an association with maternal education strengthened significantly after adjustment and became significant (*p* for trend = 0.05); men in the lowest maternal education category showed 31% higher CRP levels compared to men in the highest category (31; 95% CI: −1, 74). In women, the association with maternal education included the null (*p* = 0.2). When entered into statistical models separately, BMI and waist circumference made the most significant contribution to point estimates and significance levels in both sexes in the final models (see Discussion).

Summing up the indicators of SES at birth, lower maternal education was associated with higher CRP levels in both sexes. Notably, adjusting for indicators of obesity attenuated the association in women, whereas it amplified the association in men. Family income at birth was a risk factor in men regardless of level of adjustment, and showed no associations in women.

### Results for SES indicators at age 23 (concurrent family income and own education)

In unadjusted analyses, men in the highest family income category had 29% higher CRP levels than men in the lowest category (−29; 95% CI: −48, −3 in the lowest family income group). Women with the least education (0–4 y) had 34% higher levels (34; 95% CI: −4, 87) than those who had the most (≥12 y).

In men, after adjustment for age, race/ethnicity, indicators of SES at birth and at age 23 (model 2), higher family income in 2004 was associated with higher CRP levels, but the trend weakened slightly (*p* = 0.05). Additionally, a trend with education became apparent in men, but in the opposite direction such that those with the least education had the highest CRP levels (*p* = 0.04), although point estimates included the null. Both of these associations were fully attenuated when further adjusted for anthropometric and behavioral variables (model 3). In women, associations between indicators of SES at age 23 and CRP levels were not significant in model 2, and remained null in model 3.

To account for possible acute inflammatory conditions at the time of the blood draw, all analyses were repeated (see Statistical methods) excluding individuals with CRP levels above 10 mg/L; this included approximately 4% of men and 9% of women (Pearson et al., 2003). Results were largely consistent with findings reported above, with similar point estimates but slightly wider confidence intervals.

The interaction term (in %CRP per SES category) for [sex*family income at birth] was significant only at the highest tertile of income at 0.80 (95% CI 0.64, 1.02) for women. Similarly, the term for [sex*mother's education] was 0.74 (95% CI 0.58, 0.95) at the highest level of education (9+ years) for women. Family income at age 23 also showed interaction with sex, but only at the highest tertile at 0.77 (95% CI 0.62, 0.97) in women.

## Discussion

This study examined lifecourse indicators of SES in relation to CRP levels at age 23 in a Brazilian birth cohort. Our conceptual model included SES at birth and in young adulthood and had the advantage of prospectively-collected data, including concurrent behavioral and anthropometric variables. We found that early-life SES was significantly associated with CRP levels, independent of later-life SES but income and education had differential associations with CRP levels in men and women. Notably, adiposity accounted for the bulk of the association between lifecourse SES indicators and CRP levels.

In the current study, the effects of early SES persisted in both sexes even when adjusted for later SES. In men, a strong association between higher family income at birth and higher CRP levels was apparent when adjusted for family income at age 23 and attained (own) education. In women, higher maternal education was associated with lower CRP levels in spite of adjustment for own education and family income at age 23. These point estimates were similar in magnitude, approximately 14% and 12% per category of SES indicator in men and women, respectively. This suggests that early SES may impact CRP levels later in life independently of later SES (which may be on the causal pathway). Three recent studies in high-income countries examined similar lifecourse socioeconomic exposures in relation to CRP levels (Gimeno et al., 2008; Pollitt et al., 2007; Tabassum et al., 2008). Pollitt et al. (2007) reported “modest but consistent” associations between early-life SES and CRP in a large US cohort (lower SES associated with higher CRP levels). Tabassum et al. (2008) observed similar but more powerful associations in a large British cohort. Notably, they found that social class at birth was a stronger predictor of CRP levels at age 42 compared to social class in midlife. When simultaneously adjusted, beta coefficients (95% CI) for the associations between CRP and social class at birth were 0.07 (0.03, 0.11) and 0.08 (0.03, 0.12) in men and women, respectively, whereas the associations with social class in midlife were 0.01 (−0.03, 0.05) and 0.03 (−0.01, 0.07), respectively. Conversely, early-life SES differences in later-life CRP concentrations were not found by Gimeno et al. (2008) in a smaller and younger Finnish cohort.

It is postulated that early-life socioeconomic conditions may contribute to CVD incidence in adulthood through a number of purported mechanisms (Godfrey & Barker, 2001; Lucas, Fewtrell, & Cole, 1999). Our findings support the notion that physiological plasticity at important ‘windows’ of development permits early exposures to exert permanent effects on hormonal, metabolic, or other physiological regulatory parameters (Barker, 2003; Barker, 2004; Godfrey & Barker, 2001). In the context of developmental origins, inflammatory pathways and immunological mechanisms may also be relevant. To date, however, the specific impact of early conditions on inflammatory mechanisms has not been examined. Further research of the physiological adaptations resulting from early socioeconomic conditions is needed to elucidate the mechanisms by which social factors throughout the lifecourse may impact inflammation and inflammatory processes (Gabay & Kushner, 1999).

Higher income and lower educational attainment was associated with higher CRP levels in men and women, respectively. This pattern was more evident with early vs. later SES, lending support to our and other findings that early-life SES has a strong impact on later CRP (Tabassum et al., 2008). Data on the influence of socioeconomic indicators on CRP levels is very limited (especially outside high-income countries), but findings with respect to obesity have been described. In middle-income countries, overweight and obesity are associated with higher income and other material socioeconomic indices in men, and lower socioeconomic indices in women, as shown by previous studies and a recent systematic review by McLaren (McLaren, 2007; Mondini & Monteiro, 1998; Monteiro, Conde, & Popkin, 2001; Monteiro et al., 2004). However, when examining certain SES indicators such as education and occupation in women, associations mirror those from in high-income countries where higher education and income are linked to lower CRP levels, suggesting that “the social patterning of weight-related attributes is perhaps in transition across the development spectrum.” (McLaren 2007). Adiposity in the current cohort follows reported contemporary trends; higher-SES men but lower-SES women present higher rates of overweight and obesity whether examining income or education (Barros et al., 2006). The possibility of these and similar results being attributable to statistical artefact has been discussed but the consistency of findings is compelling (McLaren, 2007). It is notable that Sobal and Stunkard's (1989) review of the literature concluded that attitudes toward obesity in developed and developing societies seem to be “congruent with the robust associations between SES and obesity” (Sobal & Stunkard, 1989). It is possible that concurrent and early SES act through distinct mechanisms to impact health outcomes, and that these pathways interact dynamically throughout the lifecourse with socio-cultural factors related to sex and gender roles, and may be specific to the level of epidemiological transition specific to the region in study.

**Fig. 2:**
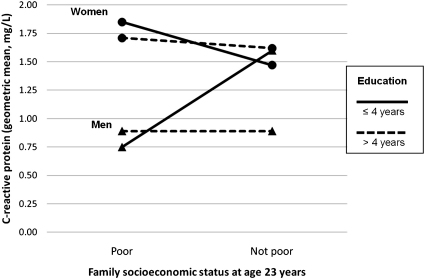
C-reactive protein concentrations in poor vs. not poor (bottom vs. top two tertiles of family income in 2004–2005) men and women with ≤4 or >4 years of education. *P*-values for interaction in men = 0.001, in women = 0.6.

Because patterns of obesity vary according to the level of socioeconomic development, it is feasible that the effects of income and education on CRP may also vary from country to country, independent of the effects of SES on obesity. Interestingly, countries in rapid transition may reflect intermediate patterns of these associations (McLaren, 2007). Our findings indicated that early and later SES indicators (with the exception of attained education) modified the effect of sex on CRP levels. This suggests that women at higher levels of SES are more protected than men in this setting. There was also a significant interaction between income and education (Fig. 2) such that men with low attained education and high concurrent income had more than twice the level of CRP as compared to other men (*p* = 0.001), suggesting that education confers benefits independent of income. Consistent with the observed and previously described differential associations of income and education between the sexes in middle-income countries, this interaction in women was not significant (*p* = 0.6). In sum, education seemed to offer a more consistent benefit than income, and both SES indicators showed sex-specific associations; with women being more protected at higher levels of SES than men.

Recent findings suggest a strong and positive association between adipose tissue and inflammation, the former mediating the latter (Mohamed-Ali et al., 1997; Panagiotakos, Pitsavos, Yannakoulia, Chrysohoou, & Stefanadis, 2005; Yudkin, Stehouwer, Emeis, & Coppack, 1999). By examining the effects of socioeconomic exposures at various levels of adjustment, we were able to show to what degree obesity accounted for the association between SES and inflammation in both sexes. In the current sample, adding behavioral and anthropometric variables to statistical models changed the magnitude of associations between SES indicators and CRP levels by approximately 7–19 percentage points, the equivalent of approximately 0.1 mg/L of CRP in men and 0.3 mg/L in women. Most of this effect was due to BMI and waist circumference, especially in women, and to a lesser extent, smoking in men (Nazmi, Oliveira et al., 2007). When adjusted for all SES lifecourse indicators (model 2), concurrent family income in men was significantly associated with CRP levels, with a seven percent mean increase in CRP levels per each increase in income category (*p* = 0.05). When adjusted for all other covariates (model 3), this association was null (*p* > 0.9). Notably, when only adiposity indicators (BMI and waist circumference) were added to model 2, a 2% mean increase per income category was observed with a non-significant *p*-value for trend (*p* = 0.6), indicating the powerful impact of obesity indicators in this association. A similar phenomenon was observed in women. Maternal education was associated with CRP levels with a mean 11% decrease per category of educational attainment in model 2 (*p* = 0.01), but the association was null when all covariates were added in model 3 (*p* = 0.2), indicating a non-significant mean decrease of 7% CRP levels per category. When only BMI and waist circumference were added to model 2, however, a mean decrease of 8% per category was found (*p* = 0.1). These findings suggest that adiposity plays an important role in the association between early and later SES and inflammatory levels. Two other studies examining lifecourse SES in relation to CRP levels found similar effects of adjusting for BMI and smoking (Pollitt et al., 2007; Tabassum et al., 2008).

Also in line with the literature describing differential impacts of SES on obesity in middle-income countries, we found that adjusting for adiposity fully attenuated the association between maternal education and CRP levels in women but strengthened the same association in men. This suggests that there may be pathways through which maternal education can act as a protective factor against elevated CRP levels, but that these effects may be offset by greater adiposity in subjects (men) born to mothers with higher SES, and only become evident after adjustment for fatness (Monteiro et al., 2004). Socio-behavioral patterns that tend to be formed early in life and act throughout the lifecourse, such as dietary and other lifestyle patterns, may influence these pathways. Social factors involved in the associations related to these paradoxical findings deserve further research, especially in the context of adiposity, dietary patterns and perceptions of body image related to sex and socioeconomic standing in middle-income countries. In both sexes, it is likely that lifecourse socioeconomic exposures impact long-term health differently in middle-income countries compared to high-income countries.

CRP levels in the current sample were similar to those from population-based samples in young adults from the USA (Ford, Giles, Mokdad, & Myers, 2004; Ford, Giles, Myers, & Mannino, 2003) and slightly lower than a New Zealand cohort of similar age (Williams, Williams, Milne, Hancox, & Poulton, 2004). There are no other population-based data of CRP distributions in young adults from a middle-income setting. Other key strengths of this study include the availability of lifecourse data on relatively young sample and an *a-priori* defined conceptual model. Additionally, we employed several levels of statistical models in order to evaluate the impact of various covariables on the outcome, aiding in interpretation.

Potential limitations of this study should be noted. Ongoing inflammatory disease status at the time of the blood draw was not assessed. However, exclusion of individuals with CRP levels greater than 10 mg/L did not markedly affect our findings. Defining clinical risk using CRP levels is made using two measurements at least two weeks apart, whereas our study relied on a single measure (Pearson et al., 2003). Notwithstanding, CRP levels have been shown to remain relatively stable over time and this likely did not impact our findings (Frohlich et al., 2002; Kayaba et al., 2000). Changes in inflammatory markers have been reported in women at different phases of the menstrual cycle, but our analysis did not account for this (Blum et al., 2005). Lifecourse analyses are not without their specific challenges, and the impact of residual confounding can be unpredictable when employing a number of related variables throughout life. For example, early and later SES indicators were moderately correlated (*r* = 0.47–0.50). Our methodological approach attempted to tease out the effects of early and later SES by analyzing these variables in three separate models. It should also be noted that family income at age 23 years might be in flux; thus our findings are applicable only to the period of young adulthood to which these analyses pertain. Finally, these cross-sectional analyses preclude implications of causality.

In sum, we found that early socioeconomic exposures exerted significant and lasting impacts on CRP levels. Our paradoxical findings for income and education in men and women warrant further investigation, especially in the context of socio-cultural and environmental conditions specific to middle-income countries that may impact adiposity, a key mediator in the SES–CRP association. Public health strategies aimed at decreasing the burden of CVD in middle-income settings, in addition to highlighting the risks associated with adult obesity, should not overlook the wide-ranging impacts of early-life social determinants.

## Figures and Tables

**Table 1:** Descriptive characteristics of the 1982 Pelotas Birth Cohort men and women at birth and at age 23 years, % or mean (SD).

	Men, *n* = 2213	Women, *n* = 2084	*P*-value
Age, mean years	22.7 (0.4)	22.7 (0.4)	–
Race/ethnicity, self-reported			0.01
White	75.0	75.9	
Black	15.4	15.9	
Mulatto	5.9	5.0	
Asian	1.5	2.0	
Indigenous	2.4	1.2	

Family income in 1982, monthly minimum wage			0.4
≤1	19.9	20.0	
1.1–3.0	49.7	49.8	
3.1–6.0	18.9	18.5	
6.1–10.0	5.9	5.9	
≥10	5.6	5.8	

Maternal education, years			0.2
0–4	32.6	33.1	
5–8	43.3	41.8	
9–11	10.8	11.2	
≥12	13.4	13.9	

Family income in 2004–2005, monthly minimum wage			<0.001
≤1	4.5	6.9	
1.1–3.0	30.8	33.9	
3.1–6.0	34.1	33.0	
6.1–10.0	16.5	13.8	
≥10	14.1	12.5	

Own education, years			<0.001
0–4	9.4	6.8	
5–8	32.4	23.5	
9–11	45.6	50.8	
≥12	12.5	18.9	

BMI, mean kg/m^2^	23.8 (4.1)	23.5 (4.7)	0.06
Centrally obese, %	10.2	26.9	<0.001
High/very high fat diet, %	64.4	63.6	0.7
Low fiber intake, %	68.9	69.4	0.8
>1 alcoholic drink per day, %	34.1	11.3	<0.001
Sedentary at leisure time, %	49.3	80.5	<0.001
High stress level, %	23.5	32.8	<0.001
C-reactive protein,^a^ median (IQR) mg/L	0.84 (0.38–1.98)	1.68 (0.64–4.37)	<0.001

Cohort members who were followed up in 2004–2005 are shown. *P*-values by chi-squared test or ANOVA. Central obesity: ≥94 cm for men and ≥80 cm for women. High stress level: ≥6 for men and ≥8 for women on SRQ-20.

a C-reactive protein (CRP) *N* = 1919 men and 1370 women (women were pregnant, *n* = 93, or using oral contraceptives, *n* = 445, at the time of the blood draw were excluded).

**Table 2:** Percent change (95% confidence interval) in C-reactive protein levels according to lifecourse socioeconomic indicators in the 1982 Pelotas Birth Cohort. Men, *n* = 1919.

Period	Socioeconomic variable	Median (IQR) CRP (mg/L)	Level of adjustment according to conceptual model			
			Unadjusted	Model 1	Model 2	Model 3
Birth 1982	Family income, monthly minimum wage^a^		0.001	<0.001	0.001	0.007
	≤1	0.73 (0.34–1.84)	−42 (−56, −21)	−47 (−62, −25)	−42 (−60, −16)	−35 (−56, −4)
	1.1–3.0	0.84 (0.39–1.86)	−32 (−48, −10)	−36 (−54, −12)	−30 (−50, −2)	−31 (−52, −1)
	3.1–6.0	0.88 (0.37–2.10)	−29 (−47, −5)	−32 (−50, −6)	−25 (−47, 4)	−25 (−47, 7)
	6.1–10.0	0.74 (0.39–1.73)	−29 (−51, 2)	−29 (−51, 2)	−26 (−49, 8)	−23 (−48, 15)
	≥10	1.02 (0.58–2.76)	0 (ref)	0 (ref)	0 (ref)	0 (ref)
	Maternal education, years^a^		0.6	0.08	0.1	0.05
	0–4	0.84 (0.39–1.84)	−9 (−25, 11)	16 (−9, 48)	19 (−8, 53)	31 (−1, 74)
	5–8	0.83 (0.37–2.06)	−11 (−26, 8)	8 (−14, 35)	12 (−11, 41)	15 (−11, 48)
	9–11	0.81 (0.35–1.88)	−15 (−33, 8)	−4 (−26, 25)	−1 (−24, 29)	8 (−19, 44)
	≥12	0.87 (0.41–2.11)	0 (ref)	0 (ref)	0 (ref)	0 (ref)

Age 23 years, 2004	Family income, monthly minimum wage		0.02	–	0.05	>0.9
	≤1	0.65 (0.37–1.74)	−29 (−48, −3)		−30 (−51, 0)	−6 (−37, 39)
	1.1–3.0	0.85 (0.35–2.09)	−21 (−35, −4)		−19 (−36, 2)	−6 (−27, 21)
	3.1–6.0	0.77 (0.37–1.84)	−22 (−36, −6)		−19 (−35, 0)	−21 (−38, 0)
	6.1–10.0	0.85 (0.38–1.98)	−17 (−33, 3)		−12 (−30, 11)	−12 (−32, 13)
	≥10	0.95 (0.47–2.15)	0 (ref)		0 (ref)	0 (ref)
	Own education, years		>0.9	–	0.04	0.7
	0–4	0.92 (0.37–1.82)	−12 (−2, 14)		17 (−14, 58)	−2 (−30, 38)
	5–8	0.85 (0.40–2.04)	−13 (−29, 6)		7 (−17, 36)	0 (−24, 32)
	9–11	0.80 (0.35–1.89)	−22 (−36, −6)		−25 (−27, 14)	−7 (−28, 19)
	≥12	0.96 (0.45–2.12)	0 (ref)		0 (ref)	0 (ref)

*P*-values for trend by linear regression. Model 1 adjusted race/ethnicity, age, family income in 1982, and maternal education. Model 2 adjusted for race/ethnicity, age, family income in 1982, maternal education, family income in 2004, and own education. Model 3 adjusted for race/ethnicity, age, family income in 1982, maternal education, family income in 2004, own education, BMI, waist circumference, smoking, fat and fiber intake, alcohol consumption, physical activity level, and stress level.

a Due to missing data, the numbers of observations for analyses were: family income in 1982 *n* = 1912; maternal education *n* = 1916.

**Table 3:** Percent change (95% confidence interval) in C-reactive protein levels according to lifecourse socioeconomic indicators in the 1982 Pelotas Birth Cohort. Women, *n* = 1370.

Period	Socioeconomic variable	Median (IQR) CRP (mg/L)	Level of adjustment according to conceptual model			
			Unadjusted	Model 1	Model 2	Model 3
Birth 1982	Family income, monthly minimum wage^a^		0.3	0.3	0.1	0.1
	≤1	1.59 (0.59–4.30)	33 (−8, 94)	−5 (−39, 48)	−19 (−48, 29)	−31 (−59, 17)
	1.1–3.0	1.69 (0.70–4.47)	45 (2, 106)	8 (−29, 63)	−6 (−39, 44)	−24 (−54, 24)
	3.1–6.0	2.01 (0.75–4.73)	60 (10, 132)	32 (−12, 99)	19 (−21, 81)	−9 (−44, 47)
	6.1–10.0	1.31 (0.49–3.03)	16 (−26, 82)	7 (−32, 69)	0 (−37, 59)	−14 (−49, 46)
	≥10	1.10 (0.50–2.65)	0 (ref)	0 (ref)	0 (ref)	0 (ref)
	Maternal education, years^a^		0.002	0.002	0.01	0.2
	0–4	1.80 (0.74–5.10)	43 (12, 81)	53 (13, 107)	35 (−2, 86)	16 (−20, 68)
	5–8	1.79 (0.66–4.48)	35 (8, 70)	40 (6, 86)	27 (−6, 71)	18 (−16, 66)
	9–11	1.54 (0.64–3.75)	18 (−12, 57)	17 (−15, 59)	11 (−19, 52)	0 (−31, 45)
	≥12	1.24 (0.52–3.17)	0 (ref)	0 (ref)	0 (ref)	0 (ref)

Age 23 years, 2004	Family income, monthly minimum wage		0.04	–	0.6	>0.9
	≤1	1.31 (0.49–4.31)	13 (−20, 59)		−8 (−38, 37)	−9 (−42, 43)
	1.1–3.0	1.99 (0.66–5.02)	42 (11, 80)		16 (−14, 56)	−9 (−35, 29)
	3.1–6.0	1.67 (0.75–4.52)	33 (5,70)		12 (−15, 49)	−11 (−36, 24)
	6.1–10.0	1.54 (0.70–4.31)	22 (−7, 62)		9 (−19, 47)	−7 (−33, 31)
	≥10	1.29 (0.50–2.81)	0 (ref)		0 (ref)	0 (ref)
	Own education, years		0.03	–	0.6	0.8
	0–4	1.67 (0.67–3.71)	34 (−4, 87)		20 (−20, 81)	4 (−36, 68)
	5–8	1.73 (0.69–4.38)	35 (8,70)		16 (−14, 58)	8 (−24, 53)
	9–11	1.77 (0.66–4.61)	34 (10,63)		19 (−6, 51)	25 (−5, 64)
	≥12	1.30 (0.54–3.25)	0 (ref)		0 (ref)	0 (ref)

*P*-values for trend by linear regression. Excludes pregnant women (*n* = 93) and those using oral contraceptive therapy (*n* = 445) at the time of the 2004–2005 cohort visit. Model 1 adjusted race/ethnicity, age, family income in 1982, and maternal education. Model 2 adjusted for race/ethnicity, age, family income in 1982, maternal education, family income in 2004, and own education. Model 3 adjusted for race/ethnicity, age, family income in 1982, maternal education, family income in 2004, own education, BMI, waist circumference, smoking, fat and fiber intake, alcohol consumption, physical activity level, stress level, and parity.

a Due to missing data, the numbers of observations for analyses were: family income in 1982 *n* = 1360; maternal education *n* = 1368.
